# SLAMF7 (CD319) on activated CD8^+^ T cells transduces environmental cues to initiate cytotoxic effector cell responses

**DOI:** 10.1038/s41418-024-01399-y

**Published:** 2024-10-10

**Authors:** Holger Lingel, Laura Fischer, Sven Remstedt, Benno Kuropka, Lars Philipsen, Irina Han, Jan-Erik Sander, Christian Freund, Aditya Arra, Monika C. Brunner-Weinzierl

**Affiliations:** 1 Department of Experimental Paediatrics, University Hospital, Otto-von-Guericke-University, Magdeburg, Germany; 2 Health Campus Immunology, Infectiology and Inflammation, Otto-von-Guericke-University, Magdeburg, Germany; 3 Institute of Chemistry and Biochemistry, Freie Universität Berlin, Berlin, Germany; 4 Multi-parametric bioimaging and cytometry (MPBIC) core facility, University Hospital, Otto-von-Guericke-University, Magdeburg, Germany; 5 Institute of Cellular and Molecular Immunology, University Hospital, Otto-von-Guericke-University, Magdeburg, Germany

**Keywords:** T cells, Acute inflammation

## Abstract

CD8^+^ T-cell responses are meticulously orchestrated processes regulated by intercellular receptor:ligand interactions. These interactions critically control the dynamics of CD8^+^ T-cell populations that is crucial to overcome threats such as viral infections or cancer. Yet, the mechanisms governing these dynamics remain incompletely elucidated. Here, we identified a hitherto unknown T-cell referred function of the self-ligating surface receptor SLAMF7 (CD319) on CD8^+^ T cells during initiation of cytotoxic T-cell responses. According to its cytotoxicity related expression on T effector cells, we found that CD8^+^ T cells could utilize SLAMF7 to transduce environmental cues into cellular interactions and information exchange. Indeed, SLAMF7 facilitated a dose-dependent formation of stable homotypic contacts that ultimately resulted in stable cell-contacts, quorum populations and commitment to expansion and differentiation. Using pull-down assays and network analyses, we identified novel SLAMF7-binding intracellular signaling molecules including the CRK, CRKL, and Nck adaptors, which are involved in T-cell contact formation and may mediate SLAMF7 functions in sensing and adhesion. Hence, providing SLAMF7 signals during antigen recognition of CD8^+^ T cells enhanced their overall magnitude, particularly in responses towards low-affinity antigens, resulting in a significant boost in their proliferation and cytotoxic capacity. Overall, we have identified and characterized a potent initiator of the cytotoxic T lymphocyte response program and revealed advanced mechanisms to improve CD8^+^ T-cell response decisions against weak viral or tumor-associated antigens, thereby strengthening our defense against such adversaries.

## Introduction

Recognition and subsequent elimination of cancerous cells by cytotoxic CD8^+^ T lymphocytes is an essential part of immune surveillance and provides the basis for T-cell mediated immune control. Due to their endogenous origin, tumor-associated antigens are typically subject to tolerance mechanisms and thus only can activate a CD8^+^ T cell repertoire of low-affinity T cells [1]. Hence, understanding and enhancing the T-cell responsiveness to these antigens to induce durable anti-tumoral responses is a major goal of cancer immunotherapy. A cascade of signals is required to achieve full-fledged cytotoxic T cells, including TCR recognition of cognate antigens, costimulatory signals, and danger cues provided by inflammatory cytokines [2, 3]. Further, priming of CD8^+^ T cells typically lasts for several days and consists of a series of events and phases resulting in populations of expanded antigen-specific T-cell clones [4]. These phases require contact-dependent information exchange [5] embedded in the formation of CD8^+^ T-cell populations that according to the Quorum hypothesis determine the outcome of a response through collective decisions rather than an individual activation of single cells [6–8]. These dynamics within CD8^+^ T-cell populations are well described and follow a remarkably reproducible and robust sequence of contact formations, involving motile kinapses and CD8-dependent stable synapses [9, 10]. Ultimately, this program could lead to ICAM-1-mediated T-cell:T-cell interactions that result in commitment to CD8^+^ T-cell expansion and differentiation [11–14]. Furthermore, these stable CD8^+^ T-cell populations facilitate the communication via intercellular IFN-γ exchange, a critical modulator of CD8^+^ T-cell differentiation [15–17].

CD8^+^ T-cell Quorum decisions are thought to increase the robustness and the quality of responses and can be regulated by intercellular receptor-interactions such as the interplay between CD28, B7 (CD80/CD86), and CTLA-4 [18]. In particular, the immuno-regulatory receptor CTLA-4 plays a pivotal role in integrating cellular contacts and the size of cell populations to regulate CD8^+^ T-cell differentiation [19]. To identify the underlying mechanisms of CTLA-4 as an immune-checkpoint, analyses of downstream targets of CTLA-4 in CD8^+^ T cells revealed novel putative regulators such as the self-ligating surface receptor SLAMF7 [20]. Because of its binding characteristics, SLAMF7 (CD319) may enable hetero- and homotypic interactions to regulate the communication and interactions between different and same types of immune cells during the complex process of an immune response [21, 22]. Various functions of SLAMF7 have been reported, such as regulation of phagocytosis and activation of macrophages, activation and degranulation of NK cells, as well as modulation of B-cell signaling [23–28]. In T cells, SLAMF7 serves as a surrogate marker for cytotoxic subsets [29, 30]. Hence, its signals may promote cytotoxicity in the cytotoxic CD4^+^ subset or reverse the defective phenotype of CD8^+^ cytotoxic T lymphocytes of patients with systemic lupus erythematosus [31, 32]; however, in cancer-specific settings its expression is also related to exhaustion or a suppressive capacity of CD8^+^ T cells [33, 34]. This versatility of effects could result from multiple SLAMF7 signaling modes depending on the intracellular molecules that bind via Src homology-2 (SH-2) domains to the two phospho-tyrosine motifs within the cytoplasmic tail of SLAMF7, such as EAT-2, SHP-1 and SHP-2, SHIP1, Csk, Fyn, or PLC-γ [24, 27].

So far, albeit SLAMF7 has become a clinically relevant target of novel cancer therapies, its function in CD8^+^ T-cells, especially during initiation of cytotoxic T-cell responses, remains elusive. In this study, we investigated the particular function of SLAMF7 after antigen-recognition of CD8^+^ T cells and characterized whether its signals promote or impede their effector differentiation.

## Methods

### Samples and T-cell activation

All experiments were performed in accordance with institutional, state, and federal guidelines. An approval has been obtained by the Clinical Research Ethics Committee of the University of Magdeburg (certificate 53/19); all donors provided written informed consent in accordance with the declaration of Helsinki (see Supplementary Methods). OVA-specific TCR^tg^ mice (OT-I), C57BL/6, and SLAMF7^−^^/−^ mice were bred under specific pathogen-free conditions in the central animal facility of the University of Magdeburg Medical Faculty (licence 42502-2-1743 Uni MD) (Magdeburg, Germany). SLAMF7^−/−^ mice (B6/JGpt-*Slamf7*^*em9Cd5012*^/Gpt) were obtained from GemPharmatech (San Diego, CA, U.S.A.). Sex- (both gender) and age-matched (age 6-12 weeks) mice were used for all experiments. All mice have been back-crossed for several generations to the C57BL/6JRj strain. Isolation of APCs or naive CD8^+^ CD44^low^ CD62L^high^ T cells from spleens, inguinal- and axillary-lymph nodes was performed with magnetic separation (Miltenyi Biotec, Bergisch Gladbach, Germany) according to the manufacturer’s instructions. Isolated WT C57BL/6 CD8^+^ T cells were seeded in 96 well flat bottom plates and activated with 0.4 µg/ml αCD3ε (#130-092-973) and 0.8 µg/ml αCD28 (#130-093-182) antibodies (both Miltenyi Biotec), coupled to microspheres composed of sulfate polysterene (ThermoFisher Scientific, Waltham, MA, U.S.A.) at a ratio of 3:2. Varying antibody concentrations are otherwise indicated. In some experiments, cells were treated with 10 µM STAT1 inhibitor (Fludarabine), 100 µM STAT4 inhibitor (Lisofylline), 5 µM cyclosporine A (all ThermoFisher Scientific), 1 µM Akt inhibitor II, 10 µM PKA inhibitor (14-22 amide), or 5 µM rottlerin (all Merck, Darmstadt, Germany). For antigen-specific activation TCR-transgenic CD8^+^ T cells from OT-1 mice were either activated with APCs (CD90 depleted splenocytes from WT or SLAMF7^−/−^ mice) or antigen presenting spheres consisting of 0.1 µg/ml peptide-pulsed MHC I complexes (DimerX I H-2K^b^:Ig fusion protein, BD Biosciences, Franklin Lakes, NJ, U.S.A.) with or without 0.5 µg/ml recombinant CD80-Fc (Biolegend, San Diego, CA, U.S.A.) coupled to microspheres, respectively. WT APCs either were pulsed with 0.2 μg/mL SIINFEKL (N4, OVA_257–264_) peptide (Invivogen, San Diego, CA, U.S.A.) or with 0.2 μg/mL V4 (SIIVFEKL) peptide (DGpeptides, Hangzhou, China), respectively. SLAMF7^−/−^ APCs were pulsed with 0.2 μg/mL V4 peptide. Antigen-presenting spheres were pulsed with 0.2 μg/mL N4, T4 (SIITFEKL) (AnaSpec, Fremont, CA, U.S.A.), or V4 peptides as indicated. To induce SLAMF7 activation 3 µg/ml αSLAMF7 antibodies (Biolegend, #152002) was added to αCD3/αCD28 on microspheres or 4 µg/ml αSLAMF7 antibodies was added to antigen presenting spheres or otherwise as indicated. An IgG1, κ isotype antibody (Biolegend, #400402) was used as control. Cells were cultured with complete medium (RPMI, with 100 U/ml Penicillin, 100 μg/ml Streptomycin (Thermo Fisher Scientific), and 10% FCS (Biochrom, Berlin, Germany) and polarized under Tc1-inducing conditions by adding 3 ng/ml IL-12 (Biolegend) or under Tc17-inducing conditions by adding 20 ng/ml IL-23, 40 ng/ml IL-6 (both Miltenyi Biotec), 2 ng/ml TGF-β (R&D Systems, Minneapolis, MN, U.S.A.), and 20 µg/ml anti-IFN-γ (Biolegend, #505834). Co-cultures of CD8^+^ T cells and SLAMF7^−/−^ or WT control APCs were cultivated with X-VIVO 15 medium (Lonza, Basel, Switzerland) supplemented with 3 ng/ml IL-12.

### MELC microscopy

Tissue sections from C57BL/6 spleen snap frozen in liquid nitrogen and stored at −80 °C. After embedding the biopsy in Tissue-Tek® O.C.T.™ Compound (Sakura Finetek, Umkirch, Germany) cryosections of 10 μm thickness were sliced and applied on silane (Merck) coated coverslips. The coverslips were stored at −20 °C. To prepare the samples for the MELC procedure, the tissue sections were rehydrated and fixed with 2% paraformaldehyde solution in PBS (Santa Cruz Biotechnology, Dallas, TX, U.S.A.) for 15 min and permeabilized with 0,2% Triton X-100 (Carl Roth, Karlsruhe, Germany), diluted in PBS, for 10 min at room temperature. Than sections were blocked with 1% Bovine Serum Albumin (Merck), diluted in PBS, for 30 min.

MELC process: Fc receptor blocking reagent (Miltenyi Biotec), propidium iodide (Merck) as a nucleic acid dye and fluorescence-labelled antibodies against the following epitopes were used: αCD3 (#100203), αCD8α (#100706), αSLAMF7 (#152006, all Biolegend). Slides with the prepared tissue sections were placed on the stage of an inverted wide-field fluorescence microscope (Leica DMi 8; HC PL APO 20x/0.80 NA lens, Leica, Wetzlar, Germany). Antibodies and PBS as washing solution were added and removed robotically by the Toponome Imaging Cycler (MelTec). By a cyclic robotic process, tissue sections were incubated with one of the given antibodies for 15 min and subsequently rinsed with PBS. Afterwards, phase contrast and fluorescence signals were imaged by the ORCA-Fusion scientific CMOS camera (C14440-20UP, 2304 × 2304 pixels, Hamamatsu, Herrsching, Germany). To delete the specific signal of the given antibody before pipetting the consecutive one, a bleaching step was performed sequentially for each marker and a post-bleaching image was taken. The fully automated process of antibody incubation, repositioning (including autofocus), fluorescence imaging and bleaching process is controlled by an adapted version of BioDecipher® Device control software (BioDecipher, Magdeburg, Germany). Fluorescence images produced by each marker were aligned pixel-wise using the corresponding phase contrast images. Images were corrected for illumination faults using flat-field correction. The post-bleaching images were subtracted from the previous fluorescence images to increase the specificity of the visualization of each marker. All processing steps were performed with MATLAB based software developed at the Institute for Molecular and Clinical Immunology at the University of Magdeburg.

### ImmunoSpot analysis and flow cytometry

For analysis of T-cell populations and proliferation, cells were labeled with CFSE (Merck) prior activation. After 24 h the number and size of formed populations were detected by ImmunoSpot S6 analyzer (CTL, Cleveland, OH, USA). Cytometric measurements were performed on a FACS-Canto II or LSRFortessa X-20 (BD Biosciences) and analyzed with FlowJo (BD Biosciences) software. Surface and intracellular molecules of CD8^+^ T cells were stained with fluorochrome-labeled antibodies. Prior to intracellular cytokine analysis, the cells were incubated with brefeldin A for 4 h. Intracellular staining was performed after the cells were fixed with 2% paraformaldehyde (Merck) in PBS for 20 min on ice and permeabilized in 0.5% saponine (Merck) in PBS/BSA. Following antibodies were used: αCD8 (#100751), αCD44 (#103039), αSLAMF7 (#152003), and αIFN-γ (#505826, all Biolegend).

### Western blot analysis and pull-down assays

Cellular extracts of activated T cells were separated on 10% SDS-PAGE gels and transferred onto nitrocellulose membranes. Blots were either probed with antibodies against phospho-Akt (S473, #9271), phospho-Akt (T308, #4056), Akt (pan, #4691), phospho-Erk1/2 (#9101), p44 MAPK (#4695), CDK6 (#3136), Cyclin D3 (#2936), β-Actin (#3700, all Cell Signaling Technology, Danvers, MA, USA), or GAPDH (Abcam, Cambridge UK), then stained with IRDye 800CW (#926-32210) and 680RD secondary antibodies (# 926-68071, both LI-COR, Lincoln, NE, U.S.A.) and visualized as well as quantified using the Odyssey scanner and software (LI-COR). Western Blot experiments were performed in two independent replicates and the original western blots are provided as supplemental information (Fig. S2).

SLAMF7 tyrosine domain Y261 and Y281 pull-down experiments were performed using the synthetic peptides CLEENAD(p)YDTIPYTE and CAPNTF(p)YSTVQIPK with the indicated tyrosine either phosphorylated or non-phosphorylated to identify interacting signaling molecules. The N-terminal cysteine in each peptide is not part of the original sequence but was added to allow for cysteine-mediated covalent peptide coupling to agarose beads according to the manual (SulfoLink Beads, Thermo Fisher Scientific). 1 × 10^7^ CD8^+^ T cells activated with 0.75 µg/ml αCD3 and 1.5 µg/ml αCD28 antibodies (both Miltenyi Biotec, Germany) coupled to microspheres were lysed and the soluble fraction of the lysate was incubated with the agarose-bound peptides. After washing with lysis buffer, bound proteins were eluted and separated by SDS-PAGE. Protein digestion and in-gel ^16^O/ ^18^O-labeling was performed as described [35]. Each peptide pull-down assay was performed in 2 independent replicates using a reverse labeling strategy. LC-MS analysis was subsequently performed by a reversed-phase capillary nanoliquid chromatography system (Ultimate 3000, Thermo Scientific) connected to an Orbitrap Q Exactive HF mass spectrometer. Identification of proteins was done by searching against the protein sequence database of mouse (Swiss Prot) and ^16^O/^18^O-based quantification was done using the Mascot Distiller software (version 2.7.1.0). Network and GO:BP enrichment analysis of identified interaction proteins was performed with the NetworkAnalyst software [36].

### Cytotoxicity assay

TCR-transgenic OT-I CD8^+^ T cells were pre-activated in X-VIVO 15 media supplemented with 3 ng/ml IL-12 for 36 h with Antigen-presenting spheres consisting of 0.1 µg/ml peptide-pulsed MHC I complexes (DimerX I) with or without 0.5 µg/ml recombinant CD80-Fc coupled to microspheres, respectively. Spheres were pulsed with 0.2 μg/mL N4, T4, or V4 antigens, respectively. Target and control cells were obtained from CD90 depleted WT splenocytes. Target cells were labeled with 10 µM CellTrace Violet (CTV, ThermoFisher Scientific) and pulsed with N4-peptide whereas control cells were labeled with 0.1 µM CTV and left unpulsed. Primed T cells then were co-cultured for 16 h with CTV^hi^ target cells or CTV^lo^ control cells at an effector to target cell ratio of 1:1. Cytotoxic activity was assessed by flow cytometry and calculated according to the ratio of target to control cells.

### Statistical analysis

Statistical analyses were performed with Prism 9 as indicated (Dotmatics, Boston, MA, U.S.A.). Sample sizes were chosen according to respective statistical tests and represent number of animals that are indicated as data points, respectively. No variances between groups could be detected by F test and normal distribution was checked by Q-Q plot and KS test. *P*-values were calculated using linear regression analysis or two-way ANOVA with Sidak’s multiple comparison test. Statistical significance is indicated as followed: *p < 0.05, **p < 0.01, ***p < 0.001, and ****p < 0.0001.

## Results

### SLAMF7 expression on CD8^+^ T cells reflects activating and environmental cues

**Fig. 1:**
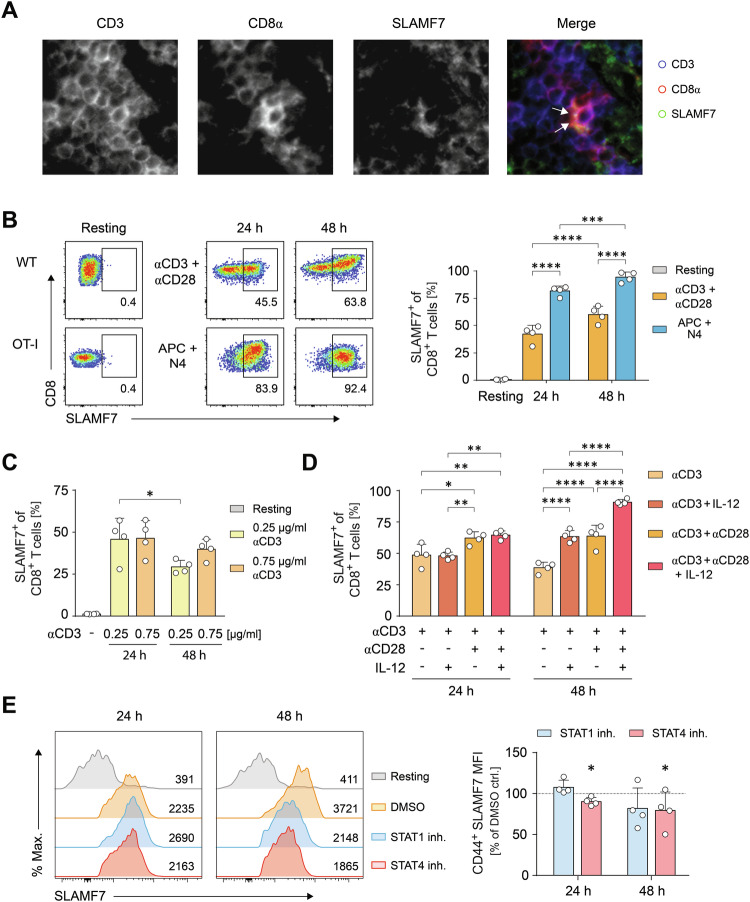
SLAMF7 expression on CD8^+^ T cells is dependent on T-cell activation signals. **A** Multi-Epitope-Ligand-Cartography (MELC) analysis of CD3 (blue), CD8α (red), and SLAMF7 (green) distribution in mouse spleen sections. Arrows indicate overlapping areas (white) in the overlay. **B** WT or OT-I CD8^+^ T cells were activated with αCD3 and αCD28 coated microspheres or OVA_257-264_ (N4) peptide pulsed APCs, respectively, or not (resting) and SLAMF7 abundancies on CD8^+^ T cells were analyzed by flow cytometry after 24 h and 48 h. **C**, **D** WT CD8^+^ T cells were activated by microspheres coated with 0.25 or 0.75 µg/ml αCD3 (**C**) or αCD3 with or without αCD28 and/or IL-12 (**D**) and SLAMF7 expression on CD8^+^ T cells was analyzed as in **B**. **E** WT CD8^+^ T cells were activated with αCD3/αCD28-coated microspheres in the presence or absence (DMSO ctrl.) of STAT1 or STAT4 inhibitor or left untreated (resting). After 24 h and 48 h SLAMF7 mean fluorescent intensities (MFI) of CD44^+^ CD8^+^ T cells were analyzed by flow cytometry. Bar graph (right panel) shows SLAMF7 expression relative to controls. Numbers indicate percentages of positive cells (**B**) or MFI (**E**). Data points represent biological replicates with mean and SD. *p < 0.05; **p < 0.01; ***p < 0.001; ****p < 0.0001; *p*-values were calculated using two-way ANOVA with Sidak’s multiple comparisons test.

To characterize SLAMF7 on CD8^+^ T cells, we initially assessed SLAMF7^+^ T cells under physiological steady-state conditions in secondary lymphoid organs, using spleen tissue sections. Multi-epitope-ligand cartography (MELC) revealed that SLAMF7 was strongly and polarized expressed along with high CD8α expression within the contact zone between CD8^+^ T cells (Fig. 1A). Since the CD8 co-receptor is necessary for the assembly of stable contacts of CD8^+^ T cells via immunological synapses, we hypothesized that SLAMF7 functions in the communication and regulation of CD8^+^ T cells during their activation. To elucidate the function of SLAMF7 in this context, we analyzed the expression of SLAMF7 after priming CD8^+^ T cells with different stimuli. Wildtype (WT) CD8^+^ T cells were stimulated using anti-CD3 and anti-CD28-coupled microspheres or Ag-specific OT-I TCR-transgenic CD8^+^ T cells were stimulated of using cognate OVA peptide loaded on APCs (Fig. 1B). As a result of physiological cell-cell-interactions, antigen-specific activated CD8^+^ T cells showed a more than 50% increased SLAMF7 expression when compared to anti-CD3 plus anti-CD28-activated T cells (Fig. 1B). Anti-CD3 stimulation alone resulted in increased SLAMF7 expression that was diminished by using lower activation strength (Fig. 1C). Both co-stimulation with anti-CD28 and the Tc1-inducing cytokine IL-12, enhanced CD3-driven expression of SLAMF7 on CTLs over time (Fig. 1D) whereas IL-2 did not show any impact (Fig. S1A). Co-stimulation with CD28 potentiated the levels of SLAMF7 on CD8^+^ T cell after both 24 h and 48 h by up to 64%, and IL-12 increased TCR-induced SLAMF7 expression after 48 h (Fig. 1D). However, the combination of three signals- TCR activation, co-stimulation by CD28, and IL-12 cytokine signaling- led to a maximum frequency of 91% SLAMF7-expressing CD8^+^ T cells (Fig. 1D). As cytokines could indirectly impinge on SLAMF7 expression via increased activation, we monitored CD44 surface expression as this receptor serves as a surrogate marker of T-cell activation [37]. Of note, CD44 expression was only enhanced after 48 h by CD28 signaling, but not affected by IL-12 (Fig. S1B).

To further investigate whether IL-12 directly promotes SLAMF7 expression via STAT4 or indirectly by IL-12-induced T-cell intrinsic IFN-γ production via STAT1, we applied chemical inhibitors of STAT1 and STAT4 during antibody-specific stimulation (Fig. 1E). To exclude any interference of the inhibitors with activation events, we analyzed SLAMF7 intensities within the activated CD44^hi^ T-cell compartment. Our experiments revealed that inhibiting STAT4, at least partly, curtailed SLAMF7 expression, whereas blocking STAT1 had no effect (Fig. 1E). In line, antibody-mediated blockade of IFN-γ did not affect SLAMF7 expression (Fig. S1C). This suggests IL-12 primarily up-regulated SLAMF7 in a direct manner.

To dissect the key signaling molecules that are responsible for the initiation of SLAMF7 expression on activating CD8^+^ T-cells, we further applied pharmacological inhibitors and again assessed SLAMF7 molecule intensities in combination with CD44 expression. Herein, a strong significant loss of SLAMF7 was detected after administration of cyclosporine A that impinges on the transcription factor NFAT, whereas inhibition of PKCθ by rottlerin also showed reduced SLAMF7, but neither the inhibition of Akt nor PKA did (Fig. S1D). Taken together, SLAMF7 expression occurs immediately after the onset of T-cell activation and is initiated in an NFAT-dependent manner by TCR triggering and further increased by CD28 co-stimulation, and IL-12 signaling. By these direct relations, SLAMF7 possesses the capability to transduce activating cues into the outcome of a cytotoxic CD8^+^ T-cell response.

### SLAMF7 expression is linked to the cytolytic CD8^+^ T-cell response program

**Fig. 2:**
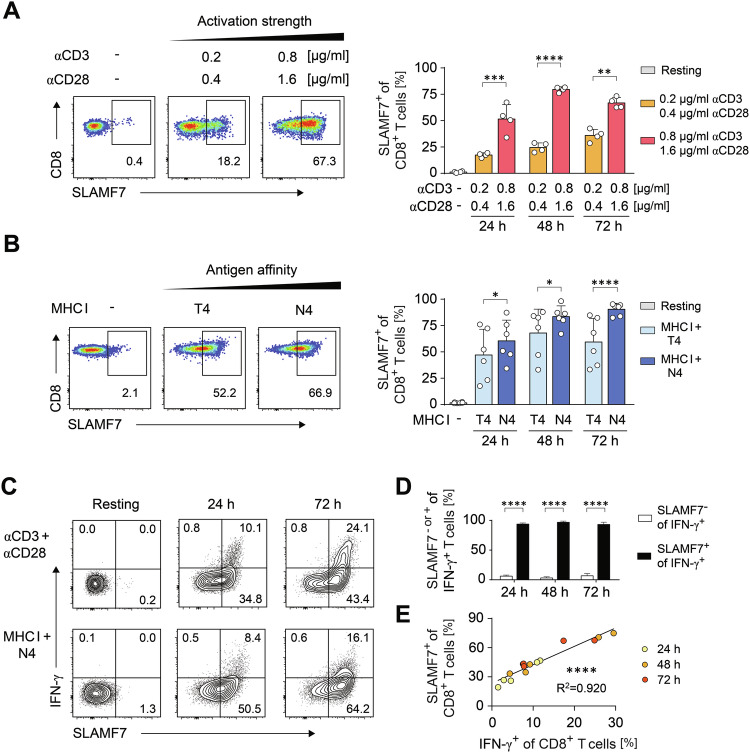
CD8^+^ T cells express SLAMF7 dependent on their activation status. **A**, **B** WT (**A**) or OT-I (**B**) CD8^+^ T cells were activated with microspheres coated with increasing concentrations of αCD3 and αCD28 (**A**) or with T4- or N4-peptide pulsed MHC I complexes in combination with CD80 (**B**), respectively, or not (resting) and SLAMF7 abundances on CD8^+^ T cells were analyzed by flow cytometry after 24 h to 72 h. **C** WT (upper panel) or OT-I TCR transgenic CD8^+^ T cells (lower panel) were activated with microspheres coated with αCD3 and αCD28 or N4-peptide pulsed MHC I complexes, respectively, or not (resting). SLAMF7 surface expression and intracellular IFN-γ of CD8^+^ T cells was analyzed by flow cytometry after 24 h to 72 h. **D** Bar graph showing SLAMF7 expression on IFN-γ positive activated WT (**C**) CD8^+^ T cells (*n* = 4); an outlier was eliminated according to Grubbs test (α = 0.01). **E** Correlation of surface SLAMF7 and intracellular IFN-γ of activated WT (**C**) CD8^+^ T cells. Numbers indicate percentages of positive cells (**A**–**C**) or correlation coefficient (R). Data points represent biological replicates with mean and SD. *p < 0.05; **p < 0.01; ***p < 0.001; ****p < 0.0001; *p*-values were calculated using two-way ANOVA with Sidak’s multiple comparisons test (**A**, **B**, **D**) or linear regression analysis (**E**).

To further delineate the role of SLAMF7 during initiation of cytotoxic CD8^+^ T-cell activation, we analyzed SLAMF7 expression at varying overall signal strengths by titrating tandem triggering of the TCR complex and co-stimulation by CD28 using dilutions of the indicated agonistic antibodies coupled to microspheres under Tc1 skewing conditions. Increasing the overall activation strength resulted in an up to four-fold increase in the frequencies of SLAMF7-expressing CD8^+^ T cells in a dose dependent manner (Fig. 2A). Since the strength of the interaction of the TCR with the peptide-MHC complex represents a critical factor for the functional outcome of the CD8^+^ T-cell response [1], we next analyzed the impact of TCR-affinity on SLAMF7 expression. Therefore we stimulated naive TCRtg-CD8^+^ T cells with cognate OVA antigen SIINFEKL (N4) compared to the low affinity OVA peptide SIITFEKL (T4) pulsed on MHC I bearing antigen-presenting spheres (Fig. 2B). Similarly, weak TCR-antigen interactions were able to induce SLAMF7 expression but cognate TCR activation further led to a significant increment (Fig. 2B). Thus, SLAMF7 expression is connected to the strength of initial cytotoxic CD8^+^ T-cell activation and thereby could function to discriminate between antigen affinities.

As cytolytic molecules such as granzyme and perforin have been found to be restricted to SLAMF7 expressing CD8^+^ T cells [32], we subsequently determined co-expression of surface SLAMF7 along with the regulatory Tc1 cytokine IFN-γ. Remarkably, both after polyclonal αCD3 and αCD28- or antigen-specific-activation, SLAMF7 was evident on over 90% of the IFN-γ-expressing effector cells (Fig. 2C, D). Importantly, there was a strong correlation (R^2^ = 0.92, *p* < 0.0001) between the frequencies of SLAMF7 surface expression and IFN-γ producers (Fig. 2E). To further investigate the connection between SLAMF7 expression and cytotoxic CD8^+^ T-cell activation, we polarized CD8^+^ T-cells by both Tc1 and Tc17 conditions, as the latter strongly prevents the induction of IFN-γ production and cytolytic functions [38]. In line, albeit being similarly activated as monitored by CD44 expression, CD8^+^ T cells polarized under Tc17 conditions failed to up-regulate SLAMF7 on their surface (Fig. S1E). Thus, activation-dependent expression of SLAMF7 on CD8^+^ T cells is hardwired within the induction of the cytotoxic CD8^+^ T-cell effector program.

### SLAMF7 promotes formation of activation-induced CD8^+^ T-cell populations

**Fig. 3:**
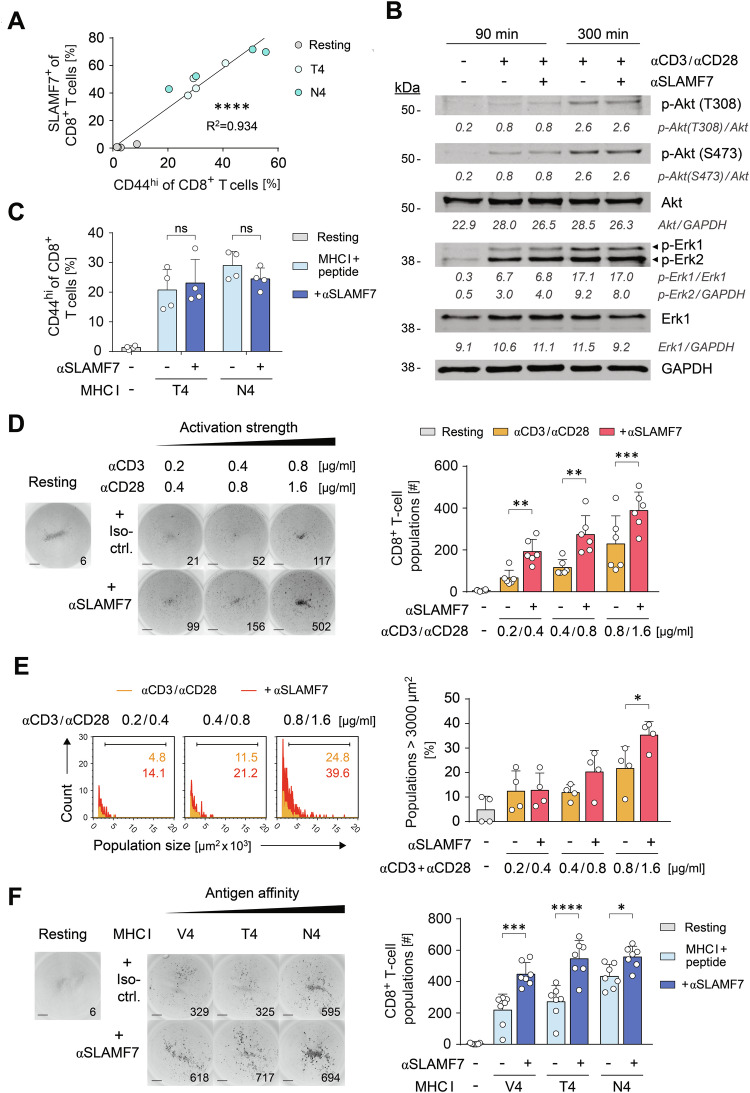
SLAMF7 facilitates initial population formation of activated CD8^+^ T cells. **A** OT-I CD8^+^ T cells were activated with microspheres coated with T4- or N4-peptide pulsed MHC I complexes in combination with CD80, or not (resting) and correlation of CD44 and SLAMF7 expression on CD8^+^ T cells was assessed by flow cytometry after 24 h. **B**, **C** WT (**B**) or OT-I (**C**) CD8^+^ T cells were activated with microspheres coated with αCD3 and αCD28 antibodies or as in **A** in combination with or without agonistic αSLAMF7 antibodies, respectively. Akt and Erk phosphorylation in non-activated WT CD8^+^ T cells or 90 and 300 min after activation was detected by Western Blot (**B**) and CD44 expression of T cells after 24 h by flow cytometry (**C**). **D** Inverted grayscale images of cell culture wells (left) and numbers (right) of CFSE-labelled population-forming WT CD8^+^ T cells activated by microspheres coated with increasing αCD3 and αCD28 antibody concentrations in combination with or without agonistic αSLAMF7 antibodies, respectively, or not (resting). Populations were counted by ImmunoSpot after 24 h, scale bar = 1 mm. **E** Size (left) and percentages of populations > 3000 µm2 (right) of WT CD8^+^ T cell populations (**D**). **F** T-cell population analysis as in **D** of OT-I CD8^+^ T cells activated by microspheres coated with CD80 and V4-, T4-, or N4-peptide pulsed MHC I complexes in combination with or without agonistic αSLAMF7 antibodies, respectively, or not (resting). Numbers indicate correlation coefficient (**A**), relative quantities of proteins (**B**), T-cell population counts (**D**, **F**), or percentages of positive cells (**E**). Data points represent biological replicates with mean and SD. ns - not significant, *p < 0.05; **p < 0.01; ***p < 0.001; ****p < 0.0001; *p*-values were calculated using two-way ANOVA with Sidak’s multiple comparisons test (**C**–**F**) or linear regression analysis (**A**).

Since SLAMF7 is immediately expressed after initial antigen-recognition of CD8^+^ T cells and its surface expression is a marker for their commitment to execute cytotoxic responses, we sought to identify the particular role of SLAMF7 in the initiation of these response programs. As these processes are defined by a sequential program that involves cellular contact dynamics, we firstly detected a strong correlation (R^2^ = 0.934, *p* < 0.0001) of SLAMF7 expression with the T-cell receptor CD44, involved in adhesion, migration, and effector function [39, 40], suggesting a possible role of SLAMF7 in this context (Fig. 3A). To identify the SLAMF7-mediated effects directly on CD8^+^ T cells, we employed an in vitro system that ensures unidirectional SLAMF7 signaling and thereby excludes possible SLAMF7 effects mediated by CD8^+^ T cells in APCs. The latter may occur in vivo by the ability of SLAMF7 to act as a self-ligand that enables bidirectional communication between interacting cells. Therefore, we activated SLAMF7 signaling by cross-linking anti-SLAMF7 antibodies that were concomitantly coupled to APC mimicking microspheres alongside TCR- and CD28-activating ligands or antibodies, respectively. The concentrations of the SLAMF7 engaging antibodies were adjusted according to previous studies [31, 32]. Further, these spheres were devoid of ligands of adhesion molecules, such as ICAM-1 or LFA-1. Because of the detected correlation of SLAMF7 expression with the activation status of CD8^+^ T cells, we firstly sought to investigate, whether SLAMF7 signals itself may enhance initial T-cell activation events. However, neither phosphorylation of Akt and Erk signaling molecules, nor expression of CD44 were increased in SLAMF7-engaged CD8^+^ T cells when compared to those T cells activated without additional SLAMF7-crosslinking antibodies (Fig. 3B, C). Nevertheless, when considering CD8^+^ T cells on the supra-cellular level, SLAMF7 signals were strikingly able to increase the formation of stable T-cell populations by two-fold independent of the TCR and CD28 activation strength (Fig. 3D). This effect mediated by SLAMF7 was not only restricted to the amount of T-cell populations visible as numbers of clusters, but it further increased the size of T-cell populations in the context of strong activating stimuli (Fig. 3E). Hence, SLAMF7 signaling caused that more T cells initiate stable contacts resulting in homotypic interactions and bigger populations. Next, we applied this mode of SLAMF7-activation to antigen-presenting spheres and found that SLAMF7-signals facilitated the formation of stable T-cell populations also in response to antigen-specific activation that was highly significant even in responses towards the lowest affinity OVA peptide SIIVFEKL (V4) (Fig. 3F). Consequently, we conclude that SLAMF7 plays an important role in the contact formation and communication between CD8^+^ T cells as indicated by MELC analysis of secondary lymphoid tissue sections (Fig. 1A). Augmenting SLAMF7 signals during initial CD8^+^ T-cell activation facilitated tight interactions of CD8^+^ T-cells that increased the amount and size of CD8^+^ T-cell populations.

### SLAMF7 function involves proteins that regulate cellular contacts

**Fig. 4:**
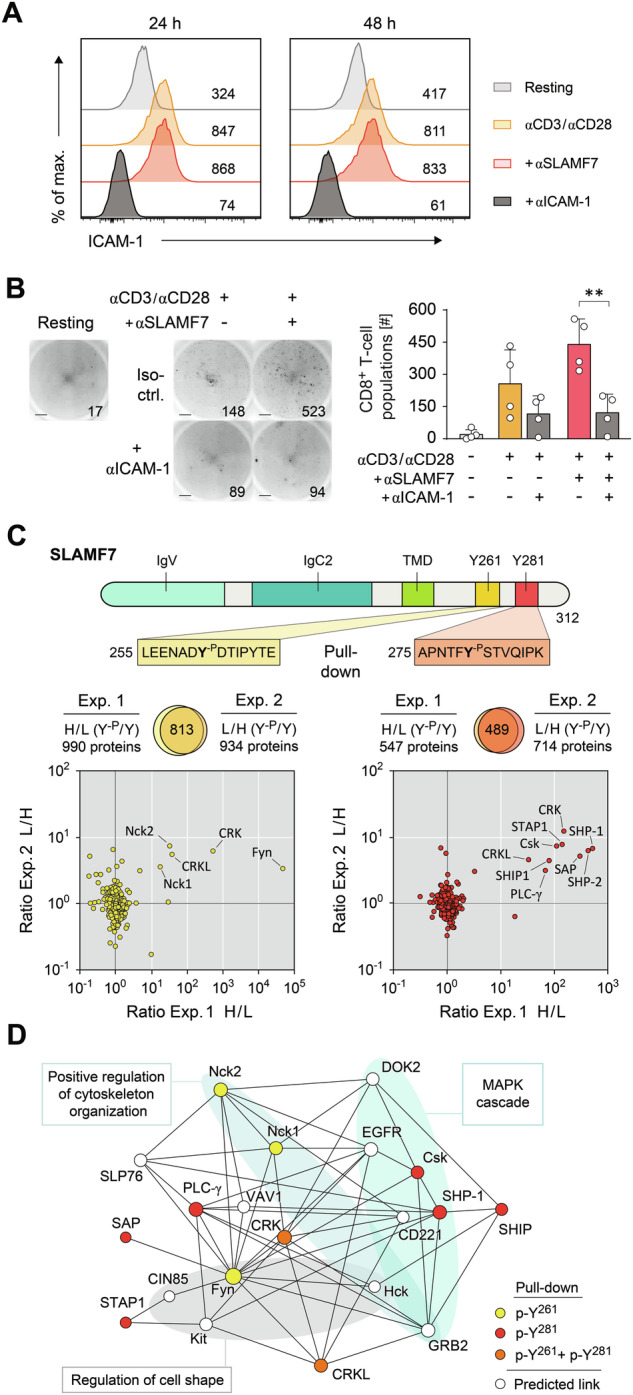
SLAMF7 signaling involves contact formation. **A** WT CD8^+^ T cells were activated by microspheres coated with αCD3 and αCD28 antibodies in combination with or without agonistic αSLAMF7 antibodies, respectively, or not (resting) and ICAM-1 was blocked by antibodies as indicated. ICAM-1 surface expression was analyzed by flow cytometry after 24 h and 48 h. **B** Inverted grayscale images of cell culture wells (left) of CD8^+^ T cells (**A**) and numbers of T-cell populations with or without ICAM-1 blockade (right). Populations were counted by ImmunoSpot after 24 h, scale bar = 1 mm. **C** Pull-down experiments using SLAMF7 synthetic peptides containing phosphorylated (Y-p) or non-phosphorylated (Y) tyrosine-motifs Y261 (yellow) or Y281 (red). Plots show the isotope ratios of all quantified proteins determined in two independent experiments with switched labeling by heavy (H) or light (L) water. Proteins with strong enrichment to the phosphorylated peptide motifs are labeled. **D** Network of possible protein interactions of identified SLAMF7-bound proteins (C) analyzed by NetworkAnalyst software [32]. Significantly (*p* < 0.001) affected functional subsets are highlighted as calculated by GO:BP enrichment analysis. Numbers indicate MFI (**A**) or T-cell population counts (**B**). Data points represent biological replicates with mean and SD. **p < 0.01; *p*-values were calculated using two-way ANOVA with Sidak’s multiple comparisons test; IgV variable immunoglobulin domain, IgC constant immunoglobulin domain, TMD transmembrane domain.

As formation of tight homotypic T-T contacts within CD8^+^ T-cell populations are dependent on ICAM-1 (CD54), we analyzed the interplay of SLAMF7 with ICAM-1. Firstly, we detected similar ICAM-1 expression levels on SLAMF7-activated and stimulated control CD8^+^ T cells and therefore excluded that SLAMF7 promotes population formation by increasing ICAM-1 abundances (Fig. 4A). Furthermore, antibody-mediated blockade of ICAM-1 during priming had no impact on SLAMF7 expression (data not shown). However, ICAM-1 blockade abolished the tight contact formation of stable T-cell populations enhanced by SLAMF7 signals (Fig. 4B). Since the activating antibody-coupled microspheres were devoid of either ICAM-1 or LFA-1, their delivered SLAMF7 signals could not impinge on the adhesion between the T cells and the microspheres itself. Therefore, the SLAMF7 engaging signals had to be transduced by the T cells to initiate and maintain tight homotypic cell contacts based on the interaction between ICAM-1 and LFA-1 on the CD8^+^ T cells.

So far, how the receptor SLAMF7 intracellularly transmits signals has been mostly derived from data using innate cytotoxic NK cells that function without variant antigen-specific receptors. CD8^+^ T cells differ from NK cells in their function of TCR sensing of antigens; therefore, we sought to identify the signaling molecules affected by SLAMF7 ligation by combining pull-down assays with mass spectrometry baiting with phosphorylated and non-phosphorylated SLAMF7 tyrosine-motifs Y261 or Y281, respectively (Fig. 4C). For discrimination, we used stable isotope labeling by light and heavy water (H_2_^16^O/H_2_^18^O) during the tryptic digestion. By plotting the heavy/light (H/L) and light/heavy (L/H) isotope ratios of proteins identified in two independent experiments with reversed labeling we found several signaling molecules to be highly enriched exclusively by the phosphorylated SLAMF7 sequences (Fig. 4C). Among the detected molecules, the adaptor proteins CRK and CRKL that are involved in T-cell adhesion and mechanosensing [41, 42], interacted with both phosphorylated SLAMF7 tyrosine domains. Further, we identified an interaction between SLAMF7 tyrosine domain p-Y281 and the TCR-related adaptors Nck1 and Nck2. Interestingly, the prototypic SLAM-adapter SAP (SH21DA) possessed the ability to associate to the SLAMF7 p-Y281 domain. Other detected signaling proteins such as EAT-2, SHP-1 and -2, SHIP1, Csk, Fyn, or PLC-γ confirm similar binding modalities compared to NK cells. To extract further information from the dataset, we applied interaction network modeling with GO:BP enrichment analysis that predicted further interacting proteins that could be allocated to GO biological processes of cell shape and cytoskeleton organization regulation as well as MAPK signaling (Fig. 4D). Network analysis of the identified p-Y261 and p-Y281 binding partners revealed the potential SLAMF7-activated signaling pathways in detail (Fig. 4D). This supports the idea that SLAMF7 actually affects how CD8^+^ T cells adhere and respond.

### SLAMF7 signals lower the threshold for CD8^+^ T cells to initiate cytotoxic responses

**Fig. 5:**
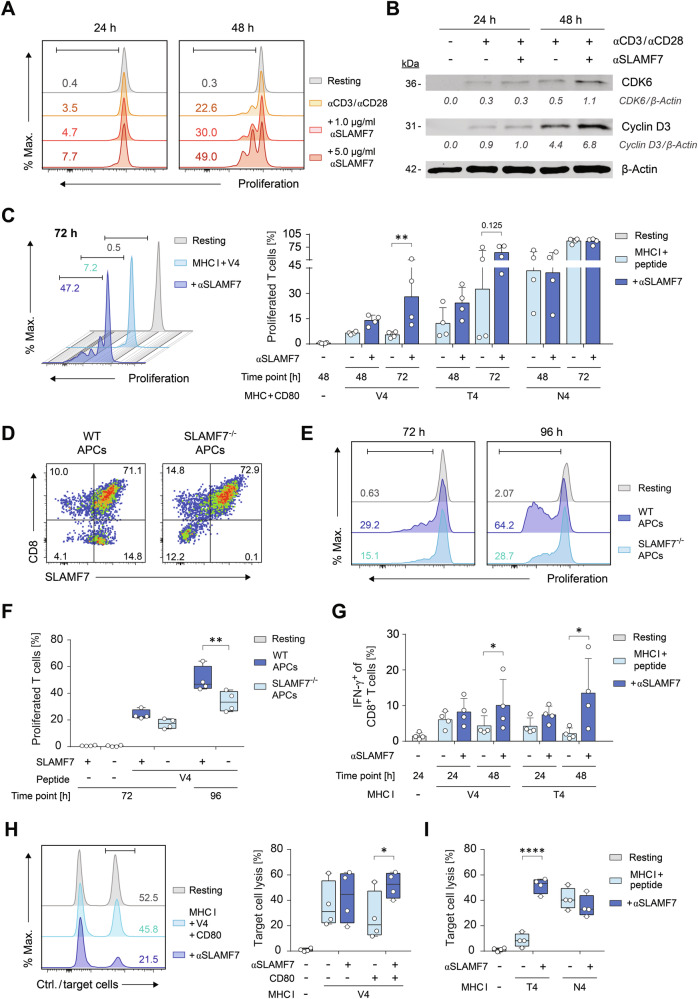
SLAMF7 signals enhance CD8^+^ T-cell responses towards low-affinity antigens. **A** WT CD8^+^ T cells were activated by microspheres coated with 0.25 µg/mL αCD3 and 0.5 µg/mL αCD28 antibodies in combination with or without increasing concentrations of agonistic αSLAMF7 antibodies, respectively, or not (resting). Proliferation of cells was monitored by CFSE dilution by flow cytometry after 24 h and 48 h. **B** Western Blot analysis of cell cycle regulators CDK6 and Cyclin D3 in WT CD8^+^ T cells at 24 h and 48 h after activation by microspheres coated with αCD3 and αCD28 antibodies in combination with or without agonistic αSLAMF7 antibodies, respectively, or not. **C** OT-I CD8^+^ T cells activated by microspheres coated with CD80 and V4-, T4-, or N4-peptide pulsed MHC I complexes in combination with or without agonistic αSLAMF7 antibodies, respectively, or not (resting). Proliferation of cells was analyzed by flow cytometry after 48 h and 72 h. **D**–**F** CFSE-labeled OT-I SLAMF7^+/+^ CD8^+^ T cells were activated by V4-peptide pulsed WT or SLAMF7^−/−^ APCs and SLAMF7 expression of co-cultured cells was determined (**D**) and proliferation of CD8^+^ T cells (**E**, **F**) was analyzed by flow cytometry after 72 h and 96 h, respectively. **G** IFN-γ production of OT-I CD8^+^ T cells activated by microspheres coated with CD80 and V4- or T4-peptide pulsed MHC I complexes in combination with or without agonistic αSLAMF7 antibodies, respectively, or not (resting). **H**, **I** Cytotoxicity assay of OT-I CD8^+^ T cells 48 h pre-activated by microspheres coated with V4- (**H**), T4-, or N4-peptide (**I**) pulsed MHC I complexes in combination with or without CD80 and agonistic αSLAMF7 antibodies, respectively, or not (resting). Cytolytic capacity was assessed by decrease of N4-pulsed high fluorescent target cells in relation to non-pulsed low fluorescent control cells. Numbers indicate percentages of positive cells (**A**, **C**–**H**) or relative quantities of proteins (**B**). Data points represent biological replicates with mean and SD (**A**, **C**, **G**) or range, interquartile, and median (**F**, **H**, **I**). *p < 0.05; **p < 0.01; ****p < 0.0001; *p*-values were calculated using two-way ANOVA with Sidak’s multiple comparisons test.

To determine whether SLAMF7 induced formation of stable contacts is a prerequisite for initiating CD8^+^ T-cell response programs, we labeled CD8^+^ T cells with CFSE to monitor proliferation prior their activation in the presence of agonistic SLAMF7 signals. Therefore, we added increasing concentrations of SLAMF7-crosslinking antibodies coupled to microspheres alongside TCR- and CD28-activating antibodies (Fig. 5A). SLAMF7 ligation by a high anti-SLAMF7 antibody concentration doubled CD3/CD28-induced proliferation 48 h after the onset of stimulation in a dose-dependent manner (Fig. 5A). These induced SLAMF7 signals led to an increased expression of cell cycle proteins that mediate the transition through G1 phase, including cyclin D3 by 55% at 48 h, as determined by western blot (Fig. 5B). In addition, levels of CDK6 that could act independently of IL-2 [43], were more than two-times higher with cross-linking of SLAMF7 than without additional SLAMF7 signals (Fig. 5B).

Next, we investigated the impact of SLAMF7 on the proliferative response to different antigen affinities as the TCR-ligand specificity crucially affects the expansion outcome. CD8^+^ T cells were stimulated using MHC I complexes, CD80, and anti-SLAMF7 antibodies (or isotype as control) coupled to microspheres mimicking antigen-presenting cells, and pulsed with lower (V4, T4) or high (N4) affinity ligands, respectively. Intriguingly, SLAMF7 signals led to a more than 5-fold increased expansion of CD8^+^ T cells 72 h after priming with very low affinity V4 antigen (Fig. 5C). However, whilst still almost doubling the proliferation in response to low affinity T4 peptide, no SLAMF7 effect was detected in the setting with high affinity N4-peptide (Fig. 5C right panel). To physiologically validate the effect of these unidirectional SLAMF7 signals that occur during the initiation of CD8^+^ T-cell responses, we activated TCR-tg CD8^+^ T cells either with V4-pulsed APCs from WT or SLAMF7^−/−^ mice and assessed the proliferative response (Fig. 5D–F). Whereas the lack of SLAMF7 on APCs during priming did not impair SLAMF7 expression of the CD8^+^ T cells (Fig. 5D), it consequently resulted in a marked decrease in CD8^+^ T-cell expansion (Fig. 5E, F).

To assess the impact of SLAMF7-mediated signals on functional outcomes of T-cell differentiation, we stimulated Ag-specific T cells using low (V4) and intermediate affinity peptides (T4). Expression of IFN-γ—a primary CD8^+^ T cell differentiation and effector cytokine of CD8^+^ T cells [17]—was analyzed and frequencies of IFN-γ producers were determined by flow cytometry. On day 2 after beginning of the stimulation, CD8^+^ T cells primed with peptides V4 or T4 showed a 2.5- to 7-fold increase in the frequency of cytokine producers, respectively, compared to cells that did not receive a SLAMF7 signal (Fig. 5G). Ultimately, to proof the enhancing effect of SLAMF7 signals in a cytotoxicity assay, we primed CD8^+^ T cells with V4, T4, or N4 pulsed antigen presenting spheres that were loaded with CD80, anti-SLAMF7, or isotype control antibodies, or a combination of both, respectively. The primed CD8^+^ T cells were then subsequently incubated with N4-pulsed high fluorescent target cells together with low fluorescent control cells. By analyzing the relative decline of target cells in comparison to control cells, we detected an up to two-fold increased cytotoxic activity in V4-primed and SLAMF7-activated CD8^+^ T cells that, of note, was dependent on CD28 signals (Fig. 5H). Similarly, a high amount of lysed target cells occurred after SLAMF7-activation with T4 priming whereas both activated control cells and SLAMF7 engaged CD8^+^ T cells showed similar cytotoxic capacities after N4 pre-activation (Fig. 5I). Thus, the observed boost by SLAMF7 in cytotoxicity was restricted to CD8^+^ T cells primed with low-affinity antigens. As these affinities typically apply to self-peptides such as tumor-associated antigens we assessed the capability of SLAMF7 signals to enhance the cytotoxic responses of human CD8^+^ T cells, activated with NY-ESO-1-pulsed HLA-A2 of antigen-presenting spheres that delivered SLAMF7 signals by anti-SLAMF7 antibodies, recombinant SLAMF7 molecules, or not [44, 45]. In line, SLAMF7 signals during initiation of CD8^+^ T-cell activation strongly increased IFN-γ and Granzyme B secretion as analyzed by ELISpot (Fig. S1F lower panel). Of note, albeit to much lower extend, this effect of SLAMF7 was also detected in CD8^+^ T-cell responses against high affinity antigens of infectious pathogens (Fig. S1F upper panel).

In summary, the SLAMF7-enhanced formation of stable CD8^+^ T-cell populations after antigen recognition and initiation of cytotoxic CD8^+^ T-cell programs profoundly improved their expansion as well as their effector functions resulting in superior responses. In particular, responses towards low affinity antigens were enhanced by SLAMF7, implicating a crucial role for SLAMF7 to regulate the decision of CD8^+^ T cells to commit to executing cytotoxic responses.

## Discussion

**Fig. 6:**
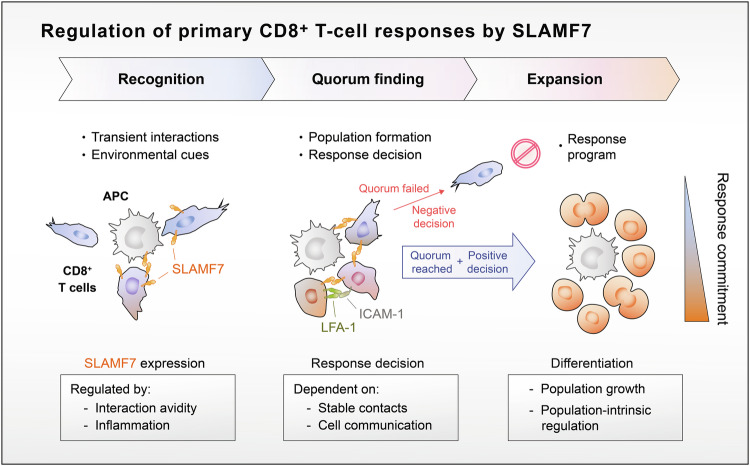
Model of SLAMF7 mediated functions during initiation of CD8^+^ T-cell responses. T-cell-initiated antigen recognition induces SLAMF7 expression on CD8^+^ T-cells dependent on activating stimuli as well as environmental cues. SLAMF7 signals then transmit this information to facilitate the formation of T-cell populations (T-cell clustering) through stable homotypic contacts that are necessary for Quorum regulation and cell communication. After a Quorum has been reached, T cells could collectively decide whether the CD8^+^ T-cell response program including clonal expansion and differentiation is initiated or not. During these processes, CD8^+^ T cells differ in their commitment to response to stimuli.

Here, we found that agonistic SLAMF7-stimuli at the early steps of initial CD8^+^ T-cell activation strongly enhanced the commitment to expand and execute cytotoxic effector functions of CD8^+^ T cells. Therefore, we not only affirm the relation of SLAMF7 as a marker for cytotoxic T cells [29, 30] but further provide a novel function of SLAMF7 and propose a coherent model that connects SLAMF7 expression as a transducer of activating and environmental cues. SLAMF7 mediated signals then support stable CD8^+^ T-cell-T-cell contacts leading to CD8^+^ T-cell populations that allow Quorum decisions and response commitment (Fig. 6). Ultimately, this response decision serves as a threshold that has to be passed in order to execute cytotoxic CD8^+^ T-cell response programs that involve expansion, effector differentiation, and population intrinsic regulatory mechanisms (Fig. 6) [8, 10–13]. Therefore, the role of SLAMF7 within initial CD8^+^ T-cell activation could be a part of a missing link that connects two activating mechanisms that do not mutually exclude each other, namely single stimuli, such as antigen avidity, co-stimulation, or inflammatory cues, with the process of T-cell quorum formation [7]. In line, this novel function of SLAMF7 could also explain the observed effect of IL-12 in augmenting of CD8^+^ T-cell population formation [11], as IL-12 signaling strongly boosted SLAMF-7 expression. Similarly inducing SLAMF7 signals promoted SLAMF7-SLAMF7 interactions also between other cell types, such as NK cells [46]. Hence, these SLAMF7-mediated stable T-cell populations increase the reliability and reproducibility of cytotoxic CD8^+^ T-cell responses by collective decisions that promote expansion and effector differentiation to determine their biological outcome [6, 18]. Importantly, these identified effects are related to the early processes within the initiation of CD8^+^ T-cell responses starting with antigen-recognition of CD8^+^ T-cells. Of note, further and later activation of CD8^+^ T cells might regulate SLAMF7 expression and function by other mediators or mechanisms.

Here, we show that the SLAMF7-mediated clustering of CD8^+^ T-cell populations depend on ICAM-1 interactions between T cells. Because of the nature of SLAMF7 to act as a self-ligand that offers a multiplicity of interactions and feedback loops, agonistic SLAMF7-mediated effects on CD8^+^ T cells observed in our study could differ from those described in SLAMF7 ko mouse models. In these models, the phenotype of the T cells primarily was strongly affected by the lack of SLAMF7 on other cell types, such as tumor-associated macrophages or B cells, giving space for further co-stimulation or signaling/inhibition by other surface molecules [26, 34]. Even though findings on the role of SLAMF7 in CD8^+^ T-cell exhaustion used sole SLAMF7 crosslinking in the absence of TCR activation [34] our results may explain these later effects: Activated cytotoxic CD8^+^ T cells express SLAMF7 and, as continuous stimulation drives exhaustion, the same cells still expressing SLAMF7 may present now an exhaustive phenotype [33, 47]. There limited responsiveness may be due to intracellular usage of a different adapter for SLAMF7 as shown for NK cells, such as the Csk or Shp phosphatases [27].

By performing pull-down experiments, putative SH-2 domain containing SLAMF7-interacting signaling molecules were identified that partially differ from those analyzed in NK cells where positive signaling relies on the adapter EAT-2 [24, 27]. The newly identified binders such as Nck, CRK and CRKL can serve to explain the observed effects. By recruiting Nck adaptors to the synapse SLAMF7 signals could modulate and lower the threshold of CD8^+^ T-cell responsiveness [48, 49]. The profound improvement of adhesion could be mediated by the CRK and CRKL adapters [50]. Furthermore, these adapters are critical sensors of inflammation that are important regulators of CD8^+^ T-cell migration and invasion [41, 42]. Moreover, to support this novel function of SLAMF7 in T-cell adhesion and communication an immunoreceptor coupling and organization motif (ICOM) has been recently described for the SLAMF7 transmembrane domain [51]. These conserved leucine zipper motifs could allow SLAMF7 to laterally interact with other transmembrane proteins to form complexes that regulate their activity. In this regard, the SLAMF7 ICOM is closely related to that of other adhesion molecules, such as cadherins, and thereby SLAMF7 could directly enhance T-cell contact formation [51]. However, SLAMF7 on CD8^+^ T cells does not merely function as a molecule that promotes adhesion. Furthermore, it combines the aspects of both cellular contacts and communication and acts as a co-transducer that integrates antigenic and environmental cues in the context of T-cell populations into the outcome of a CD8^+^ T-cell response.

Intriguingly, the findings of this study are especially related to the process of CD8^+^ T-cell responses towards low-affinity antigens. By augmenting SLAMF7 signals during CD8^+^ T-cell priming, we were able to induce an effective response to the very low affinity V4 antigen that is about 700-fold less potent than the adequate N4 peptide [52] and towards the tumor-associated self-antigen NY-ESO-1 that is shared among different tumor entities [45]. By enhancing the T-cell population dynamics the subsequent IFN-γ exchange might promote the expansion of the low avidity T cells [53]. This relation could also be important to defend viral antigens and widen the window of response when the antigen availability is restricted and only few host cells are exploited for viral replication. Further, targeting of SLAMF7 on CD8^+^ T cells could serve as a mean to regulate the formation of ICAM-1-LFA-1 dependent CTL populations within the tumor tissue [54], and by enrichment of tumor-specific CD8^+^ T cells, SLAMF7 signals could improve the tumor control.

As SLAMF7 and its function within the initiation of CD8^+^ T-cell responses could be addressed by immune-checkpoint inhibitors, such as CTLA-4 and PD-1, it has been shown that these inhibitory checkpoint receptors strongly impinge on contact formation and migration behavior of CD8^+^ T-cells, too [55, 56]. Especially CTLA-4 expression on CD8^+^ T cells is tightly connected to ICAM-1 as well as homotypic T-cell interactions and functions as a critical regulator of T-cell population dynamics that are decisive for the differentiation and the magnitude of CD8^+^ T-cell responses [11, 18, 19]. Therefore, as SLAMF7 is targeted by CTLA-4 [20] we presume that CTLA-4 could utilize SLAMF7 mediated interactions between CD8^+^ T cells to communicate via IFN-γ exchange that ultimately increase the robustness of T-cell response dynamics. In this regard, these population-intrinsic feedback loops serve to adjust the overall outcome of a T-cell response [18] and may function as a mechanism to compensate inappropriate activation of a small fraction of T-cells within the population, that e.g. individually differ in the expression of an immune-checkpoint receptor. Therefore, these regulatory circuits might also explain the physiological outcome of the T-cell response in mixed bone marrow chimeras of CTLA-4 competent and deficient mice by Quorum sensing at the population level [57].

The results of this study support a novel role for SLAMF7 on CD8^+^ T cells, which upon expression renders these cells to be committed to exert their cytotoxic effector response. Therefore, SLAMF7-expressing T cells may be good candidates for autologous cell therapy for tumor rejection, as it potentially enhances collective cell decisions to improve CD8^+^ T-cell responses against weak antigens. In this regard, targeting SLAMF7 with agonistic signals could serve as an advanced mechanism to induce or at least strongly support CD8^+^ T-cell responses during immunotherapy.

## Supplementary information

Supplementary Information

## Data Availability

The datasets generated during and/or analyzed during the current study are available from the corresponding author on reasonable request.
