# Supplementary material for: SLAMF7 (CD319) on activated CD8+ T cells transduces environmental cues to initiate cytotoxic effector cell responses
Source: Cell Death Differ. 2024 Oct 10;32(3):561–72. doi: 10.1038/s41418-024-01399-y (PMC11893764; doi:10.1038/s41418-024-01399-y)
Supplement: Supplementary file 1 — Supplementary Information [file 41418_2024_1399_MOESM1_ESM.pdf]

SUPPLEMENTARY INFORMATION

Supplementary Materials and Methods

Activation of $CD8^+$ T cells and ELISpot analysis

Healthy adult peripheral blood mononuclear cells (PBMCs) were obtained by density gradient centrifugation using Pancoll (PAN BioTech, Aidenach, Germany) of blood from leukocyte reduction filters (Sepacell RZ-2000; Asahi Kasei Medical, Tokyo, Japan) provided by the German Red Cross (DRK, Dessau, Germany). This study was formerly approved by the Ethics Board (vote 53/19) of the University of Magdeburg. All patients provided written informed consent in accordance with the declaration of Helsinki.

HLA-A2-typed $CD8^+$ T cells were isolated from PBMCs using MACS technology (CD8 MicroBeads, Miltenyi Biotec, Bergisch Gladbach, Germany) according to the manufacturer's protocol. $CD8^+$ T cells then were activated by antigen-presenting spheres consisting of peptide-pulsed microspheres composed of sulfate polysterene (ThermoFisher Scientific, Waltham, MA, U.S.A.) coated with 2 µg/ml HLA-A2:Ig (DimerX I, BD Biosciences, Franklin Lakes, NJ, U.S.A.) in combination with or without 3 µg/ml anti-SLAMF7 antibodies (clone 162.1, Biolegend, San Diego, CA, U.S.A.), or 4 µg/ml recombinant SLAMF7-Fc protein (R&D Systems, Minneapolis, MN, U.S.A.). Protein amounts were equalized by an isotype control antibody (Biolegend), respectively. Spheres were pulsed with 1 µg/ml NY-ESO-1 PepMix (JPT Peptide Technologies, Berlin, Germany) or peptide epitopes for different infectious agents (CEFX MHC-I, JPT Peptide Technologies) and used at a ratio cells:spheres of 3:2. $CD8^+$ T cells were cultured in X-VIVO 15 medium (Lonza Bioscience, Walkersville, MD, U.S.A) supplemented with 10 ng/ml human IL-12 (Proteintech, Rosemont, IL, USA). 10 ng/ml IL-2 (Proteintech) was added on day 3.

After 6 days, $CD8^+$ T cells were washed with PBS, 1:4 diluted in serum-free CTL-Test™ Medium (CTL Europe, Rutesheim, Germany) supplemented with 2 mM L-glutamin (Thermo Fisher Scientific, Waltham, MA, U.S.A), transferred onto a pre-coated PVDF multiscreen plate (Merck Millipore, Darmstadt, Germany), and an ImmunoSpot® IFN-γ/Granzyme B Double-Color ELISpot assay (CTL Europe) was conducted for 24 h according to the manufacturer's protocol. In brief, wells were pre-coated with anti-IFN-γ and anti-Granzyme B capture antibodies (CTL Europe) and thoroughly washed after incubation of $CD8^+$ T cells. Secreted IFN-γ and Granzyme B molecules were detected by anti-IFN-γ FITC and biotinylated anti-Granzyme B antibodies and after incubation with tertiary anti-FITC-HRP and Streptavidin-AP, ELISpots were developed with cognate substrates (all CTL Europe). Spots then were counted by an ImmunoSpot ELISpot reader (CTL Europe) and after subtraction of background calculated to spot forming cells per 10^5 $CD8^+$ T cells.

Suppl. Figure 1

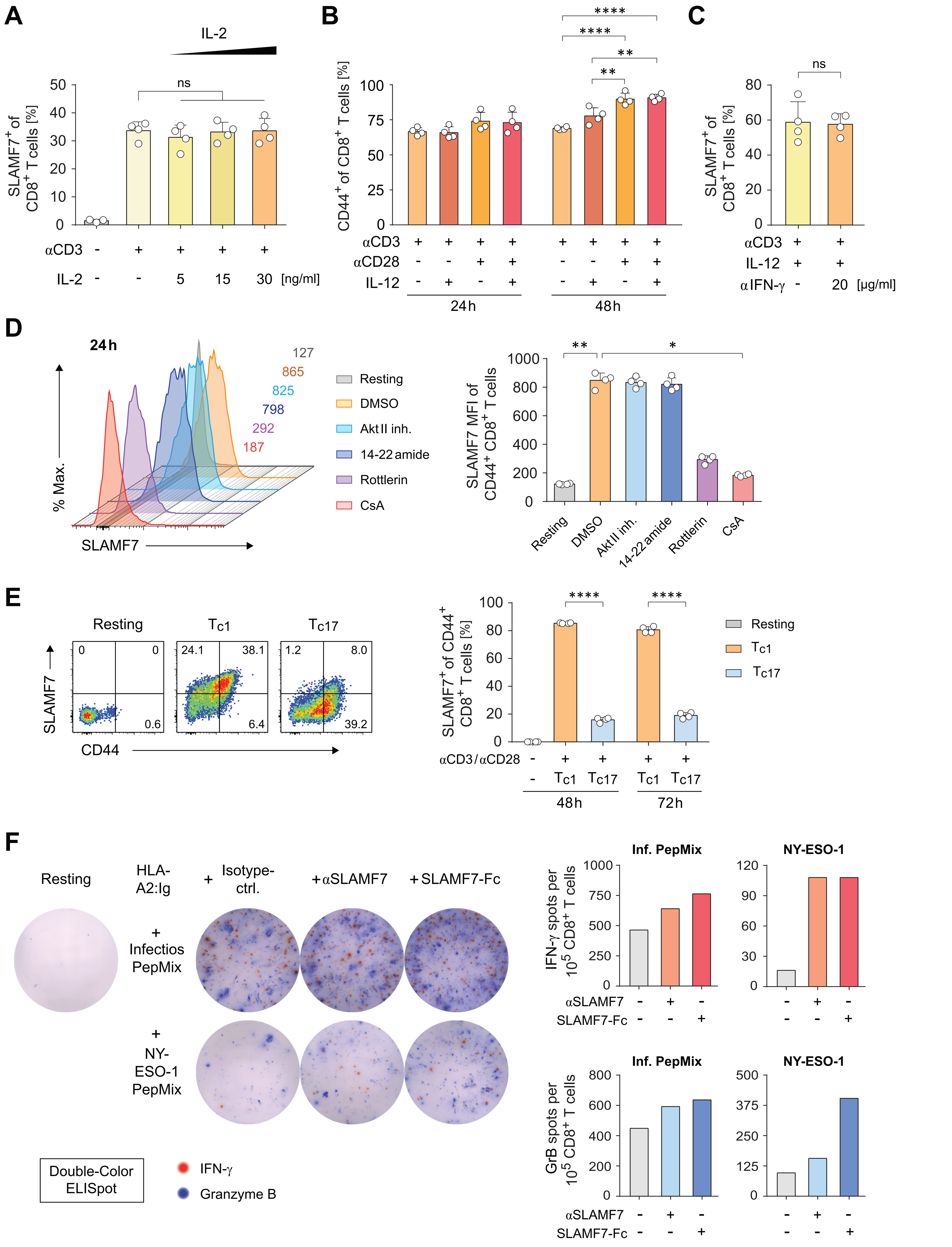

Fig. S1. Regulation of SLAMF7 expression in CD8⁺ T cells and SLAMF7-improved CD8⁺ T-cell responses to tumor-associated or infections antigens. (A-C) WT CD8⁺ T cells were activated by microspheres coated with αCD3 or αCD3 and αCD28 antibodies as indicated in combination with or without increasing IL-2 concentrations (A), IL-12 (B,C), and αIFN-γ blocking antibodies (C), respectively. After 24 h (B) and/or 48 h (A-C), surface expression of SLAMF7 (A,C) or CD44 (B) were detected by flow cytometry. **(D)** WT CD8⁺ T cells activated by microspheres coated with αCD3 and αCD28 antibodies were treated with pharmacological inhibitors of Akt (Akt II), PKA (14-22 amide), PKC-θ (rottlerin), or calcineurin-NFAT (cyclosporine A, CsA) and SLAMF7 MFI of CD44⁺ CD8⁺ T cells were analyzed by flow cytometry after 24 h. **(E)** WT CD8⁺ T cells were activated as in (D) with Tc1- or Tc17-polarizing conditions, or not (resting). SLAMF7 and CD44 surface expression was detected by flow cytometry after 48 h (left panel) and 72 h and percentages of SLAMF7 on CD44⁺ CD8⁺ T cells were analyzed (right panel). **(F)** IFN-γ/Granzyme B double-color ELISpot analysis of human CD8⁺ T-cell responses to peptide mixes of infectious pathogens or tumor-associated antigen NY-ESO-1 on antigen presenting spheres coated with recombinant HLA-A2:Ig and SLAMF7-Fc, αSLAMF7, or isotype control antibodies, respectively or not (resting). An ELISpot assay was conducted 6 d after activation for 24 h and secreted IFN-γ (red) or Granzyme B (GrB, blue) in spots were counted by an ELISpot reader. Numbers indicate mean fluorescence intensities (MFI) (D) or percentages of positive cells (E). Data points represent biological replicates with mean and SD. * $p < 0.05$; ** $p < 0.01$; **** $p < 0.0001$; p-values were calculated using one-way ANOVA (Friedmann) with Dunn's multiple comparisons test (A,D) Wilcoxon test (C), or or two-way ANOVA (B,E) with Sidak's multiple comparisons test.

Suppl. Figure 2

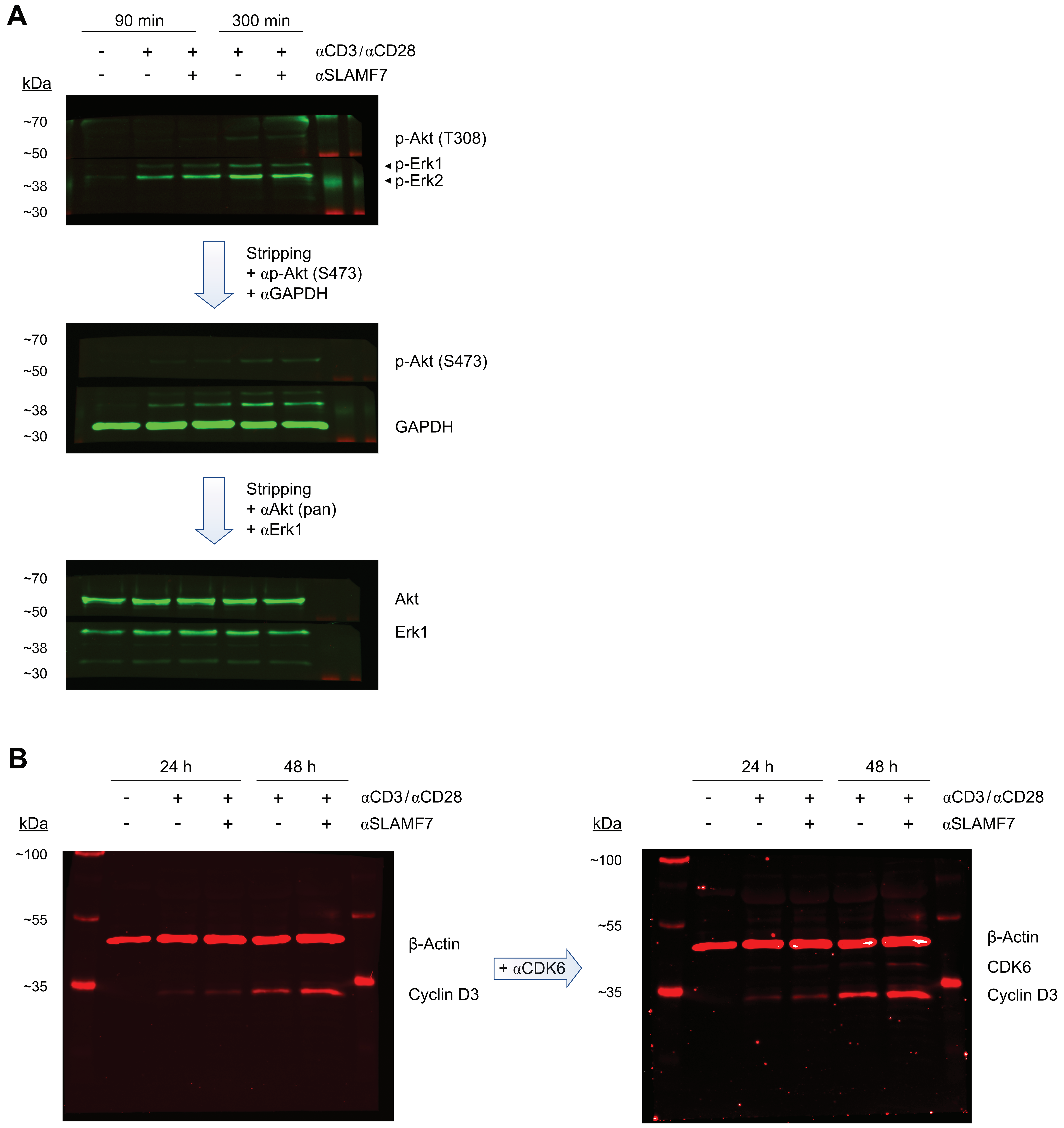

Fig. S2. LI-COR scans of Western Blot membranes. (A) Akt and Erk phosphorylation in non-activated WT CD8+ T cells or 90 and 300 min after activation by microspheres coated with αCD3 and αCD28 antibodies in combination with or without agonistic α SLAMF7 antibodies, respectively, as detected by Western Blot (Fig. 3B). Membranes were probed, stripped, and subsequently reprobed as indicated. **(B)** Western Blot analysis of cell cycle regulators CDK6 and Cyclin D3 in WT CD8+ T cells at 24h and 48h after activation by microspheres coated with αCD3 and αCD28 antibodies in combination with or without agonistic αSLAMF7 antibodies, respectively, or not (Fig. 5C). Membrane was first probed with αβ-Actin and αCyclin D3 and subsequently reprobed with αCDK6. Numbers indicate molecular weights of the respective protein standard (ladder).
